# *Rhodiola crenulata* polysaccharide alleviates dextran sulfate sodium-induced ulcerative colitis in mice by repairing the intestinal barrier and regulating the intestinal microecology

**DOI:** 10.3389/fphar.2025.1519038

**Published:** 2025-03-26

**Authors:** Jia Lv, Xinyu Kong, Wenjun Liu, Zhenzhen Su, Fengshou Luo, Fengtai Suo, Zhenzhong Wang, Liang Cao, Zhongqiu Liu, Mengxuan Li, Wei Xiao

**Affiliations:** ^1^ International Institute for Translational Chinese Medicine, Guangzhou University of Chinese Medicine, Guangzhou, China; ^2^ State Key Laboratory on Technologies for Chinese Medicine Pharmaceutical Process Control and Intelligent Manufacture, Jiangsu Kanion Pharmaceutical Co., Ltd., Lianyungang, China

**Keywords:** polysaccharide, structure characterization, ulcerative colitis, gut microbiota, short chain fatty acids

## Abstract

Polysaccharides, vital biological macromolecules ubiquitous in organisms, have garnered attention as potential therapeutic candidates for ulcerative colitis (UC). However, the therapeutic potential of *Rhodiola crenulata* polysaccharides (RCP) in UC remains largely unexplored. The RCP was prepared by boiling water extraction, 80% alcohol precipitation, membrane separation, and D101 macroporous resin purification. The monosaccharide composition of RCP (Mw = 67.848 kDa) includes mannose, rhamnose, glucuronic acid, galacturonic acid, glucose, galactose, and arabinose, with a molar ratio of 0.22:1:0.07:7.03:2.88:0.64:4.12. *In vivo* experiments have shown that RCP can improve DSS induced weight loss in UC mice, decrease disease activity index (DAI), alleviate histopathological changes in colon tissue, and suppress the levels of pro-inflammatory cytokine IL-6 and MPO activity. Immunohistochemical results showed that essential tight junction proteins such as occludin, claudin1, and ZO-1 were upregulated, improving the integrity of the intestinal barrier. Importantly, RCP regulated the abundance of the intestinal microbiota by reducing the Firmicutes-to-Bacteroidetes ratio (F/B), increasing beneficial bacteria such as Muribaculaceae and *Bifidobacterium*, decreasing harmful bacteria including Erysipelotrichaceae, *Faecalibaculum*, *Lachnospiraceae_unclassified*, *Parabacteroides*, and *Ruminiclostridium_9*. Additionally, it enhanced the restoration of acetic acid, propionic acid, isovaleric acid, and valeric acid to maintain intestinal SCFA levels, thereby restoring the intestinal microecology. Therefore, RCP has excellent therapeutic effects on UC and is worthy of further drug development and clinical treatment.

## 1 Introduction

Ulcerative colitis (UC) is a chronic, nonspecific colorectal inflammation, mainly characterized by intestinal pathological mucosal damage, ulceration, colon curtail, diarrhea, and bloody stools (Peyrin-Biroulet et al., 2015). Globally, the occurrence and prevalence of UC have been steadily escalating over time, gradually emerging as a significant challenge to global public health. The highest prevalence rates of UC have been reported in Europe (505 per 100,000), Canada (248 per 100,000), and the USA (214 per 100,000) (Ungaro et al., 2017). Since 1990, with the increasing westernization and urbanization of many newly industrialized countries, the incidence rate of UC has continued to rise (Ng et al., 2017). In recent years, the number of patients with UC in China has gradually increased. According to statistics, the incidence rate of UC in China is 1.18 per 100,000 person years, with a predominance in males (sex ratio: 1.29) (Li et al., 2017). The key risk factors for UC include genetics, environmental factors, autoimmunity and gut microbiota (Gajendran et al., 2019). The current conventional treatment methods, such as 5-aminosalicylate (5-ASA), corticosteroids, immunosuppressants, anti-tumor necrosis factor (TNF) therapies, and other therapies, focus on treating and controlling disease symptoms, often with serious adverse reactions, poor clinical efficacy, and high recurrence rates (Bressler et al., 2015; Feuerstein et al., 2020). Therefore, it is urgent to find safe and effective new drugs to alleviate UC.

Polysaccharides are polyhydroxy polymers containing aldehyde or carbonyl groups formed by dehydration of 10 or more monosaccharide molecules to form glycosidic bonds. From the perspective of bioinformatics, polysaccharides are continuously synthesized and metabolized through the synergistic effects of at least hundreds of proteins. Polysaccharides carry much more structural information than proteins, nucleic acids, and lipids (Lowe and Marth, 2003; Zhang, 2010). Therefore, polysaccharides have important energy, structure, and biological functions in all living organisms. Polysaccharides extracted from natural sources have attracted extensive attention in recent years owing to their safety, accessibility and potent anti-inflammatory activities (Xie et al., 2023). Tamarind xyloglucan and *Atractylodes macrocephala* polysaccharides are used for the prevention and treatment of enteritis (Periasamy et al., 2018; Yang et al., 2022); the polysaccharides derived from *Aphanothece sacrum* and *Polyporus umbellatus* exhibit protective effects against steatohepatitis (Goto et al., 2021; Gao et al., 2023); the polysaccharides from Enteromorpha prolifera and *Saposhnikovia divaricata* have anti-arthritic effects (Wang et al., 2022; Sun et al., 2023); as well as polysaccharides extracted from Laminaria japonica and Stigma maydis protect against nephritis injury (Li et al., 2020; Wang et al., 2023). These studies indicate that screening new drugs for the treatment of UC from polysaccharides is a promising avenue.

*Rhodiola crenulata* (Hook.f. et Thoms.) H. Ohba is a plant belonging to family Crassulaceae, whose roots and rhizomes are generally used as a medicine (Chinese Pharmacopoeia Commission, 2020). *R*. *crenulata* has a long history of being applied to regulate immune function in traditional Chinese medicine. The efficacy of *R*. *crenulata* was recorded in the classic Tibetan medical classic “Four Medical Tantras” as early as the 12th century A.D (Yuandan, 1983). Polysaccharides have been considered as the main components for immunomodulatory activities exerted by natural products (Yin et al., 2019). Polysaccharides are one of the most critical components of *R*. *crenulata*, mainly consisting of glucose (Glc), galactose (Gal), galacturonic acid (GalA), mannose (Man), rhamnose (Rha), and arabinose (Ara) (Song et al., 2008). Many studies have shown that Rhodiola polysaccharides possess various activities, including liver protection (Xu et al., 2018), frozen sperm protection (Yang et al., 2016), immune regulation (Cai et al., 2012; Cheng, 2019), antitumor activity (Wu et al., 2023), hypoglycemic and lipid-lowering effects (Yingming, 2017), as well as antioxidant activity (Xu et al., 2018). Wang et al. reported on the treatment of UC with *R*. *crenulata* extract and analyzed the small molecule components of the extract but did not mention the polysaccharide components (Wang et al., 2021). However, there are currently no relevant research reports on the therapeutic activity of Rhodiola polysaccharides for UC.

Intestinal barrier disruption and intestinal microbiota are critical factors for the development of UC. The tight junction (TJ) proteins, comprising occludin, claudins, and zonula occludens (ZO), are crucial for the maintenance of epithelial barrier integrity (Stevenson et al., 1986; Furuse et al., 1993). Alterations in the intestinal flora of UC patients have been observed, characterized by decreased microbial diversity and aberrant flora structure. Numerous polysaccharides derived from natural plants have demonstrated their capacity to modulate the intestinal microbiota (Hou et al., 2020). Short-chain fatty acids (SCFAs) are byproducts of bacterial breakdown of dietary fibers. SCFAs provide energy for the colonic epithelial cells and regulate immune homeostasis, which are helpful to treat UC (Rose et al., 2007). This study aimed to explore the role of *R. crenulata* polysaccharide (RCP) in the treatment of UC, thereby providing a theoretical foundation for the research and development of new UC drugs.

## 2 Materials and methods

### 2.1 Materials and reagents

The dry root of *R. crenulata* (Hook.f. et Thoms.) H. Ohba was purchased from Bozhou Materia Medica Market (Bozhou, China) and identified by Professor Zhenzhong Wang from Jiangsu Kanion Pharmaceutical Co., Ltd. (Nanjing, China). Monosaccharide references (mannose, ribose [Rib], rhamnose, glucose, glucuronic acid [GlcA], galactose, galacturonic acid, xylose [Xyl], arabinose and fucose [Fuc]) were purchased from Solarbio (Beijing, China). DSS (M.W 40,000 Da) was obtained from MACKLIN (Shanghai, China). Salicylazosulfapyridine (SASP) was sourced from Shanghai Fuda Pharmaceutical Co., Ltd. (Shanghai, China), while antibodies targeting ZO-1, occludin, and claudin1 were acquired from Abcam (Cambridge, UK). ELISA kits for interleukin-6 (IL-6) were procured from Thermo Fisher (MA, USA), and a myeloperoxidase (MPO) activity assay kit was provided by Wuhan Saipei Biotechnology Co., Ltd. (Wuhan, China).

### 2.2 Animals

C57BL/6 male mice, aged 8–10 weeks and weighing 22 ± 2 g, were obtained from the Experimental Animal Center of Hangzhou Medical College (Hangzhou, China), with the license No.: SCXK (Zhe) 2019–0002. All animals were housed in a controlled environment with an ambient temperature of 22–24 °C and relative humidity maintained between 50% and 60%, subjected to a 12-h light/dark cycle, with continuous access to food and water. All animal experimentation protocols received approval from the Institutional Animal Care and Use Committee (IACUC) of Jiangsu Kanion Pharmaceutical Co., Ltd. (Approval NO. 2023101712).

### 2.3 Extraction and purification of RCP

The dry rhizomes of 500 g *R*. *crenulata* were coarsely powdered and extracted twice by refluxing with distilled water (1:10, w/v) at 100 °C for 2 h each time. The combined extracts were filtered and centrifuged to remove precipitates. The supernatant was concentrated to an appropriate volume, and ethanol was added in a fourfold volume to precipitate the supernatant, which was then left overnight at 4 C. After centrifugation, the precipitate was redissolved in distilled water, and the solution was separated by the multifunctional membrane experimental equipment RNM-18G (Shandong Bona Biotechnology Group Co., Ltd., Jinan, China) with cut-off of 10 kDa. Subsequently, the solution was passed through D101 macroporous resin to remove pigments and proteins. Finally, the obtained fraction was collected and freeze-dried to yield a light brown crude polysaccharide from *R*. *crenulata*.

### 2.4 Characterization of RCP

#### 2.4.1 Determination of the total carbohydrate content

The total carbohydrate concentration of RCP was analyzed utilizing the phenol-sulfuric acid method, referencing glucose as the standard (DuBois et al., 1956).

#### 2.4.2 Determination of molecular weight distribution

The molecular weight of RCP was measured using size exclusion chromatography (SEC). The analysis was conducted using two columns (Shodex OHpak SB-805 and 803, 300 × 8 mm) connected in series, integrated with an Thermo U3000 HPLC system equipped with a multi-angle laser light scattering and differential refractive index detection (MALLS-RI). An aqueous solution containing 0.1 M NaNO_3_ was used as the mobile phase, flowing steadily at 0.6 mL/min. Data were acquired and processed using ASTRA6.1 software (Hu et al., 2017; Cao et al., 2024).

#### 2.4.3 Analysis of monosaccharide content

Monosaccharide composition of RCP was detected by Thermo Fisher Ulitimate 3000 HPLC, employing 1-phenyl-3-methyl-5-pyrazolone (PMP) for pre-column derivatization, following the procedure outlined by Luo et al. (2022).

### 2.5 Construction of animal model and treatment

**FIGURE 1:**
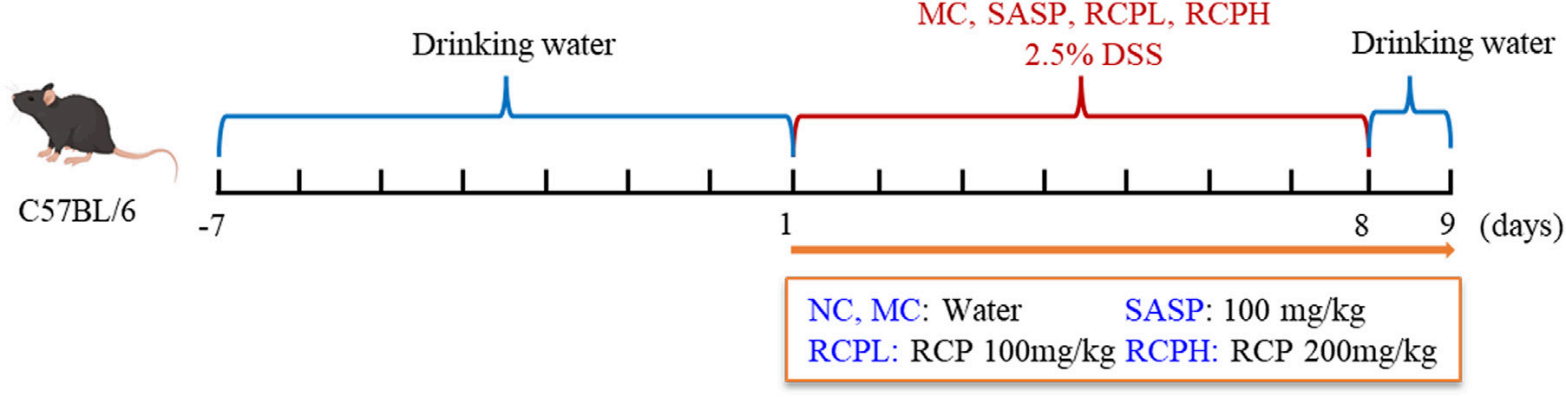
Model establishment of colitis induced by DSS.

After a 7-day acclimation period, the mice were randomly allocated to five groups: normal control (NC), model control (MC), salicylazosulfapyridine (SASP), low-dose RCP (RCPL), and high-dose RCP (RCPH), with 10 animals per group. While the NC group received regular water, the other groups were given 2.5% DSS in their drinking water for 7 days to induce an UC model, with the DSS solution replaced on days first, third, and fifth. As shown in Figure 1, during the modeling process, medication was administered simultaneously. The RCPL and RCPH groups received RCP doses of 100 mg/kg and 200 mg/kg, respectively, while the SASP group was given 100 mg/kg. The NC and MC groups were given pure water. All treatments were administered *via* gastric lavage for 8 days.

### 2.6 Assessment of disease activity index (DAI)

During the experiment, the body weights of the mice were recorded daily, and their fecal morphology and presence of blood in feces were observed and scored according to specific criteria, with reference to the standards outlined by Xu et al. (2020).

### 2.7 Sample collection

After 8 days of administration, mice were fasted for 12 h and allowed to drink water freely. Perform orbital vein blood collection on mice at day ninth. Blood samples were collected and then centrifuged at 3000 rpm for 15 min to separate serum, which was subsequently stored at −80°C. Following blood collection, the mice were euthanized *via* cervical dislocation. Colon length from cecum to anus was measured and recorded. Colonic and cecal contents were harvested and frozen at −80°C for later analysis of microbiota and SCFAs. Colon tissues were rinsed in PBS and divided into three sections: Distal sections were fixed in 4% paraformaldehyde for histological examination with H&E staining, while the other two sections were kept at −80°C for further experiments.

### 2.8 Biochemical assays

IL-6 Cytokine level in blood serum was assayed using enzyme-linked immunosorbent assay (ELISA). Additionally, colon tissues were homogenized in a pre-cooled PBS solution. The level of MPO in the colon homogenate was measured in accordance with the manufacturer’s instructions.

### 2.9 Immunohistochemical analysis

The expression of TJ-associated proteins in colon was assessed using immunohistochemical analysis (Cui et al., 2021). Panoramic scanning images were captured through NanoZoomer-S210 slice scanner and read using NDP software. The results were quantified by ImageJ software.

### 2.10 Sequencing of gut microbiota

Samples of mouse colonic contents were submitted to Sanshu Biotechnology Co., Ltd. (Shanghai, China) for 16S rRNA gene sequencing analysis. Quality control of the experimental results, Operational Taxonomic Units (OTUs) clustering, and annotation were performed.

### 2.11 Determination of SCFAs in caecal contents

An exact amount of colon content was meticulously weighed and subsequently treated with the addition of 50 μL of a 30% phosphoric acid solution, immediately followed by the introduction of 300 μL of acetone solution. This admixture was thoroughly homogenized for a duration of 3 min, thereafter subjected to centrifugation at 12,000 rpm for 10 min,and the supernatant was collected. Depending on the actual situation, the supernatant was diluted several times before the content of SCFAs was determined using gas chromatography (Agilent 7820, USA) coupled with a quadrupole mass spectrometry detection system (Agilent 5977, USA).

### 2.12 Statistical analysis

All statistical evaluations were conducted using GraphPad Prism 9. Analysis of variance (ANOVA) was applied, with the data expressed as mean ± standard deviation (SD). Statistical significance was set at *p* < 0.05, while *p* < 0.01 indicated highly significant differences.

## 3 Results

### 3.1 RCP characterization analysis

**FIGURE 2:**
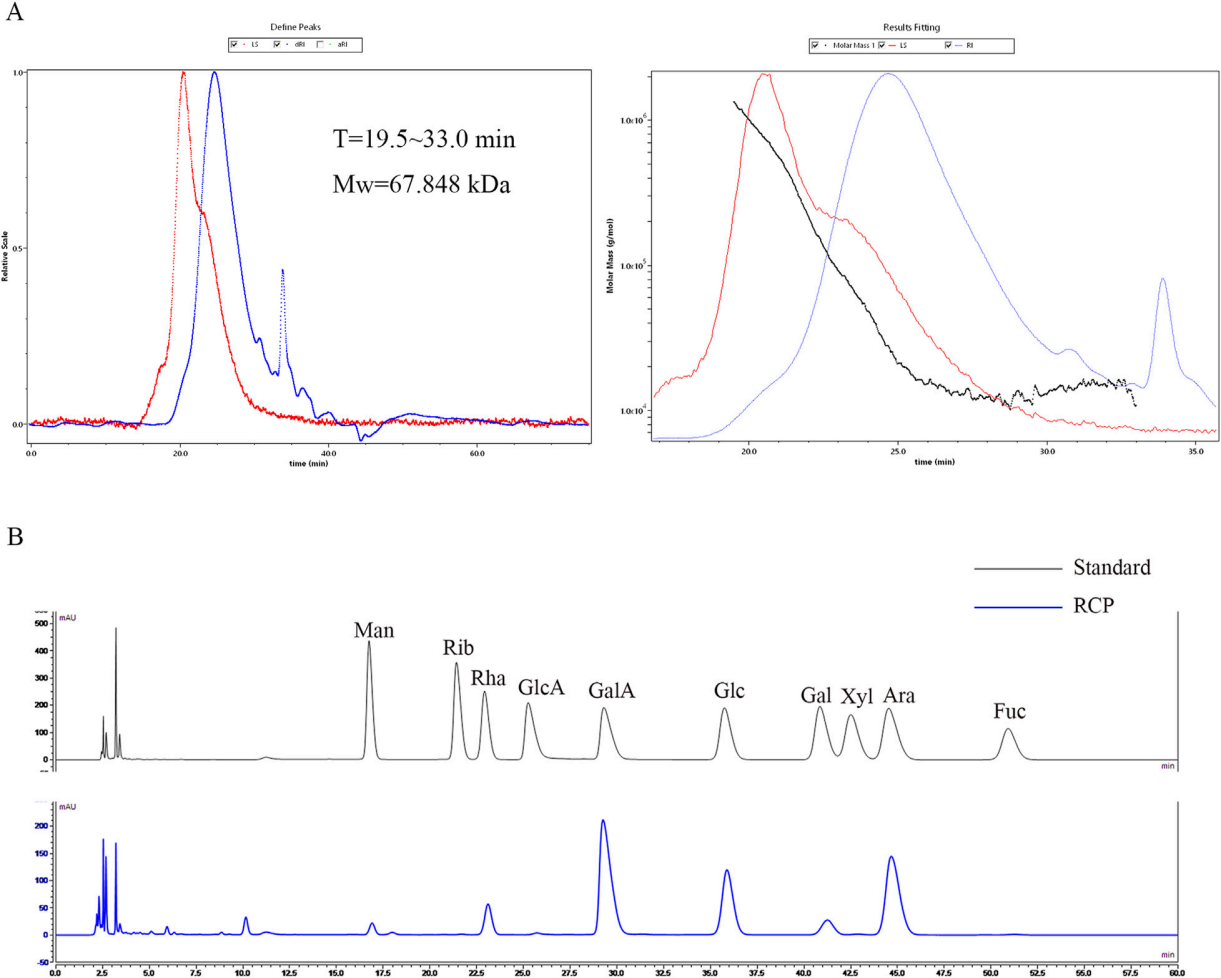
The characterization of RCP. **(A)** The SEC-MALLS-RI of RCP. **(B)** Chromatogram of pre-column PMP derivatization by HPLC for mixed monosaccharide standards and RCP.

The proportion of polysaccharides in RCP purified from the water extract of *R. crenulata* was 58.15% ± 0.016%, the standard curve was y = 9.3195x + 0.0281, *R* ^2^ = 0.9915. There is a peak detected by MALLS-RI, indicating that the molecular weight of RCP is relatively concentrated in aqueous solution. The calculated number average molecular weight (Mn) of RCP is 20.834 kDa, weight average molecular weight (Mw) is 67.848 kDa, peak molecular weight (Mp) is 27.942 kDa, and polydispersity index (Mw/Mn) is 3.257. The monosaccharide composition was assessed using pre-column PMP derivatization followed by HPLC (Figure 2B), revealing that RCP comprised Man, Rha, GlcA, GalA, Glc, Gal, and Ara, with a relative molar ratio of 0.22: 1: 0.07: 7.03: 2.88: 0.64: 4.12, indicating that RCP was a type of heteropolysaccharide. Among RCP, the content of GalA was the highest, followed by Ara.

### 3.2 RCP ameliorates colitis induced by DSS

**FIGURE 3:**
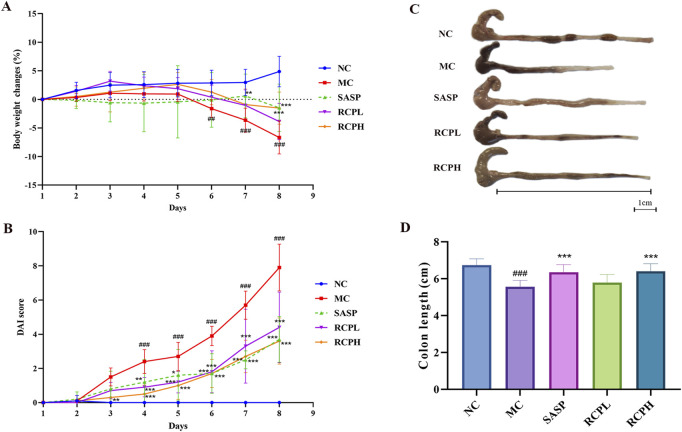
RCP’s effect on DSS-induced UC in mice. **(A)** Body weight changes over time. **(B)** Graph of DAI scores. **(C, D)** Images of colon length. Data are presented as mean ± SD (*n* = 10). Statistical comparisons: ^##^ *p* < 0.01, ^###^ *p* < 0.001 vs NC group, and *p < 0.05, **p < 0.01, ***p < 0.001 vs MC group.

The body weight of mice experienced a notable decrease after DSS induction. However, the trend of weight loss was significantly reversed through RCP treatment (Figure 3A). DSS could also lead to a substantial increase in DAI, whereas, RCPL and RCPH treatments effectively mitigated the elevation of DAI, and this improvement was positively correlated with the administered dose (Figure 3B). To more accurately assess the severity of colitis and its impact on the mice, we specifically measured the colon length of mice in each group (Figure 3C). Experimental results revealed that in the MC group, the colon length of mice was significantly shorter than that of the NC group, visually reflecting the colonic tissue damage caused by colitis. Nevertheless, in the groups treated with RCPL and RCPH, the shortening of colon length was alleviated to varying degrees, demonstrating that RCP treatment significantly improves the colon shortening induced by colitis (Figure 3D).

**FIGURE 4:**
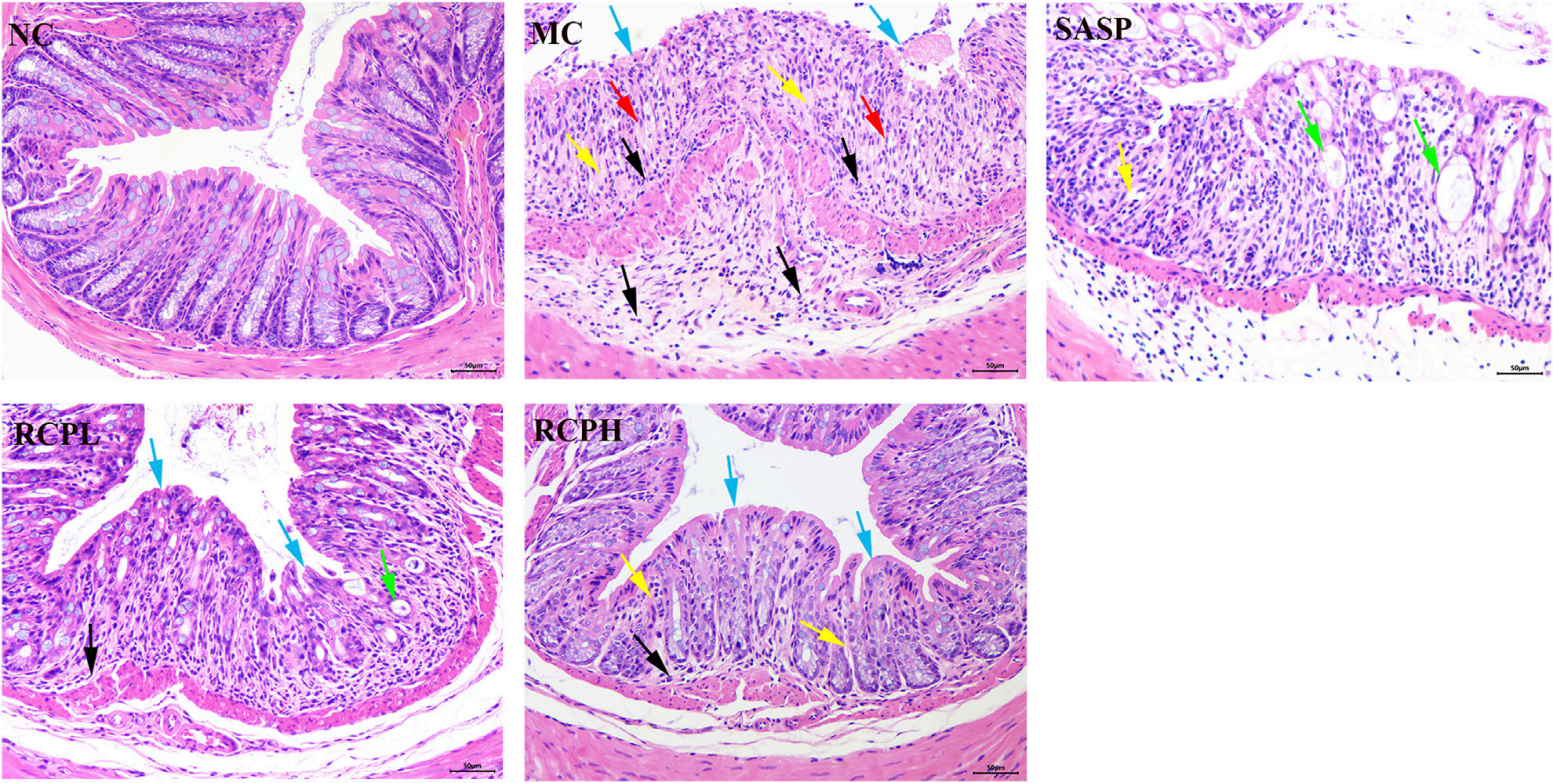
H&E stains of mice colon tissue (200×).

HE staining results of the colon are shown in Figure 4. In the NC group, the mice had an intact intestinal mucosal layer, abundant intestinal glands with compact arrangement, normal morphology of epithelial cells, abundant goblet cells, normal morphology of the muscle layer, and no obvious tissue abnormalities. In the MC group, the mice showed colonic mucosal epithelial necrosis (blue arrows), crypts forming ulcers (yellow arrows), goblet cell reduction and replacement with proliferation of connective tissue (red arrows), more inflammatory cell infiltration (black arrows). In the SASP mice, small-scale crypt loss (yellow arrows), intestinal dilation, and glandular epithelium thinning were observed (green arrow). In the RCPL group, the lesions were significantly reduced compared with the MC group. The colonic mucosal epithelial integrity of the mice was relatively intact (blue arrows), inflammation was reduced (black arrow), and a small number of intestinal gland cavities were slightly dilated (green arrow). In the RCPH group, the mice exhibited intact mucosa (blue arrows), mild inflammation (black arrow), and restored crypt structure (yellow arrows), the lesions were significantly reduced compared with the MC group.

The results indicate that the DSS-induced mouse model of UC is successful and can be used to study the interventional effect of RCP on UC. RCP significantly protects the integrity of colonic mucosa and intestinal glandular cells, prevents abnormal connective tissue proliferation, and reduces inflammation. The intensity of its effects is positively correlated with the oral dose.

### 3.3 RCP decreases inflammatory cytokine production and MPO level

**FIGURE 5:**
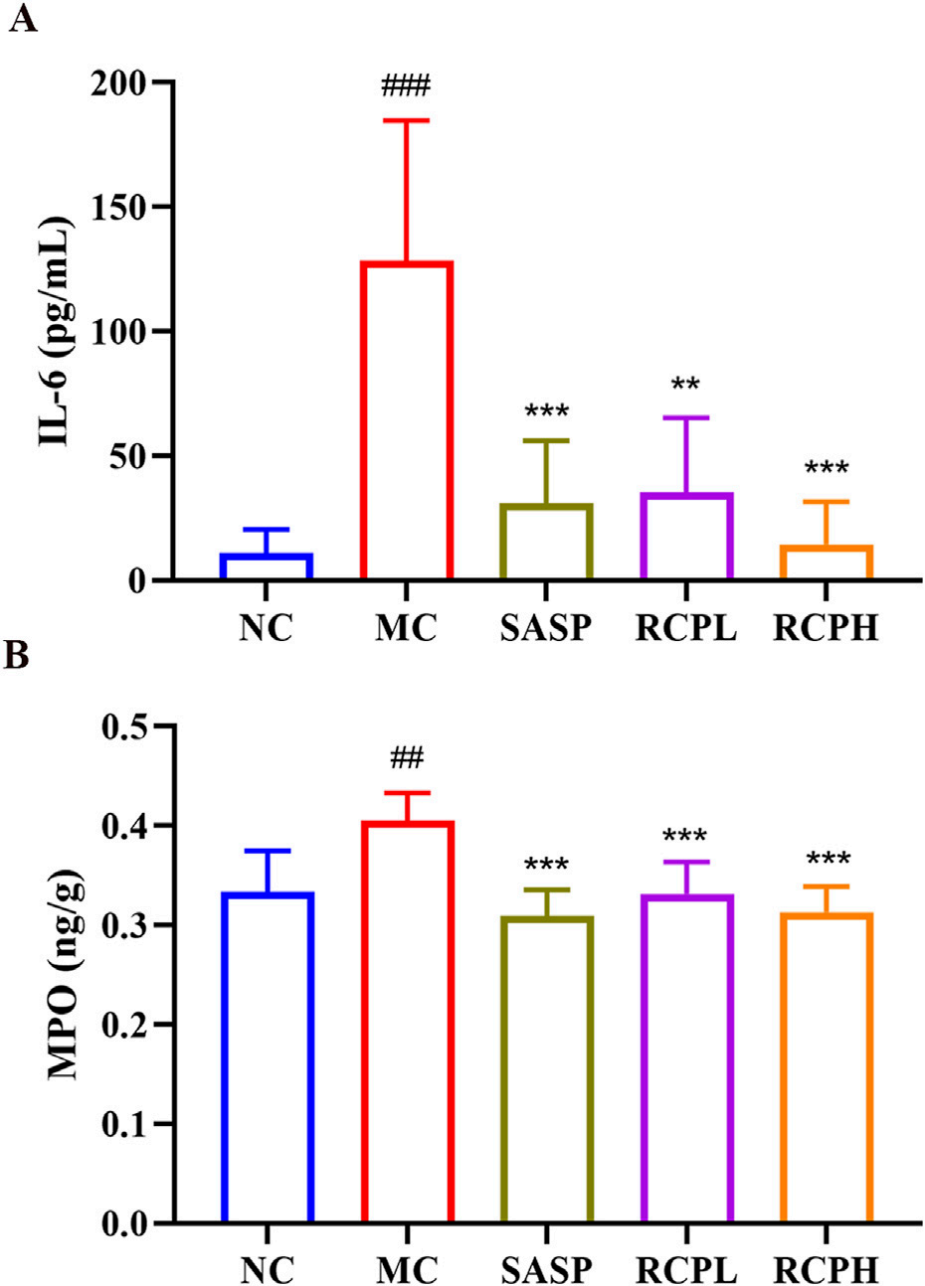
RCP’s effect on pro-inflammatory cytokines and MPO activity (*n* = 6). **(A)** Serum levels of IL-6. **(B)** Colonic tissue MPO activity. Statistical comparisons: ^###^ *p* < 0.001, ^##^ *p* < 0.01 *versus* NC group. **p < 0.01, ***p < 0.001 *versus* MC group.

As shown in Figure 5A, a significant increase in the protein expression of the IL-6 pro-inflammatory cytokine was observed in the MC group. However, RCP significantly inhibited this elevation in expression. We determined the expressions of MPO in the colon homogenate of each group using ELISA. In Figure 5B, it was observed that the MC group had higher concentrations of MPO, while the RCP treatment groups could decrease the activity of MPO. This indicates that RCP could alleviate the inflammatory response in UC, enabling it to return to a normal state gradually.

### 3.4 RCP can restore the intestinal barrier function

**FIGURE 6:**
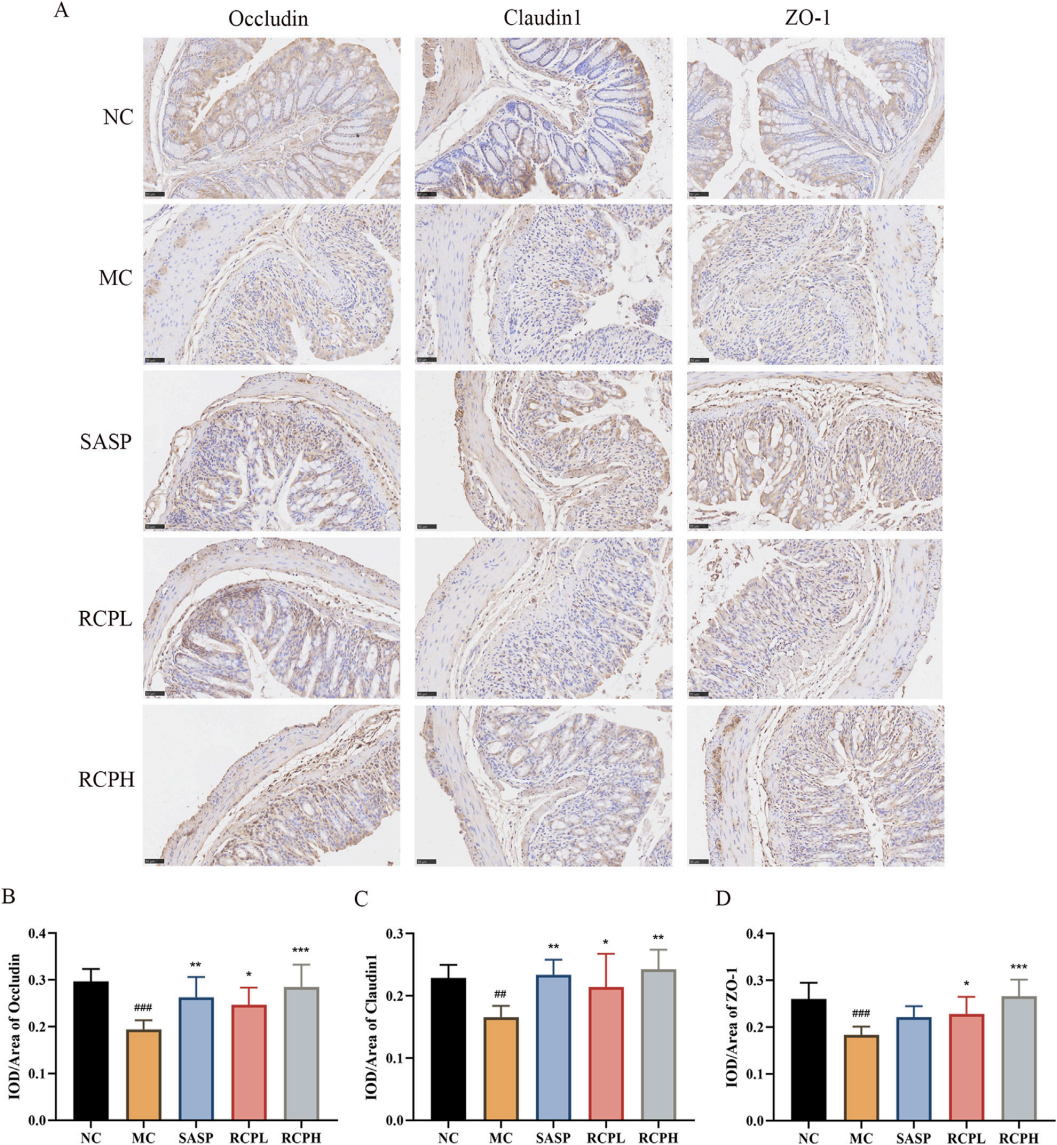
Impact of RCP on TJ protein levels. **(A)** Immunohistochemical staining of TJ proteins in colonic tissue (400×). **(B–D)** TJ proteins immunohistochemical analysis. ^##^ *p* < 0.01, ^###^ *p* < 0.001 *versus* NC group, and *p < 0.05, **p < 0.01, ***p < 0.001 *versus* MC group.

Immunohistochemical analysis revealed a reduction in the expression of TJ proteins such as occludin, claudin 1, and ZO-1 in the MC group, indicating impaired intestinal barrier function. In contrast, RCP treatment notably enhanced the expression of TJ proteins compared to DSS-induced colitis (Figure 6). This result is consistent with the HE staining result. Therefore, RCP could exert a therapeutic effect on UC by repairing the intestinal barrier through increased expression of TJ proteins.

### 3.5 RCPH alleviates DSS-induced gut microbiota

**FIGURE 7:**
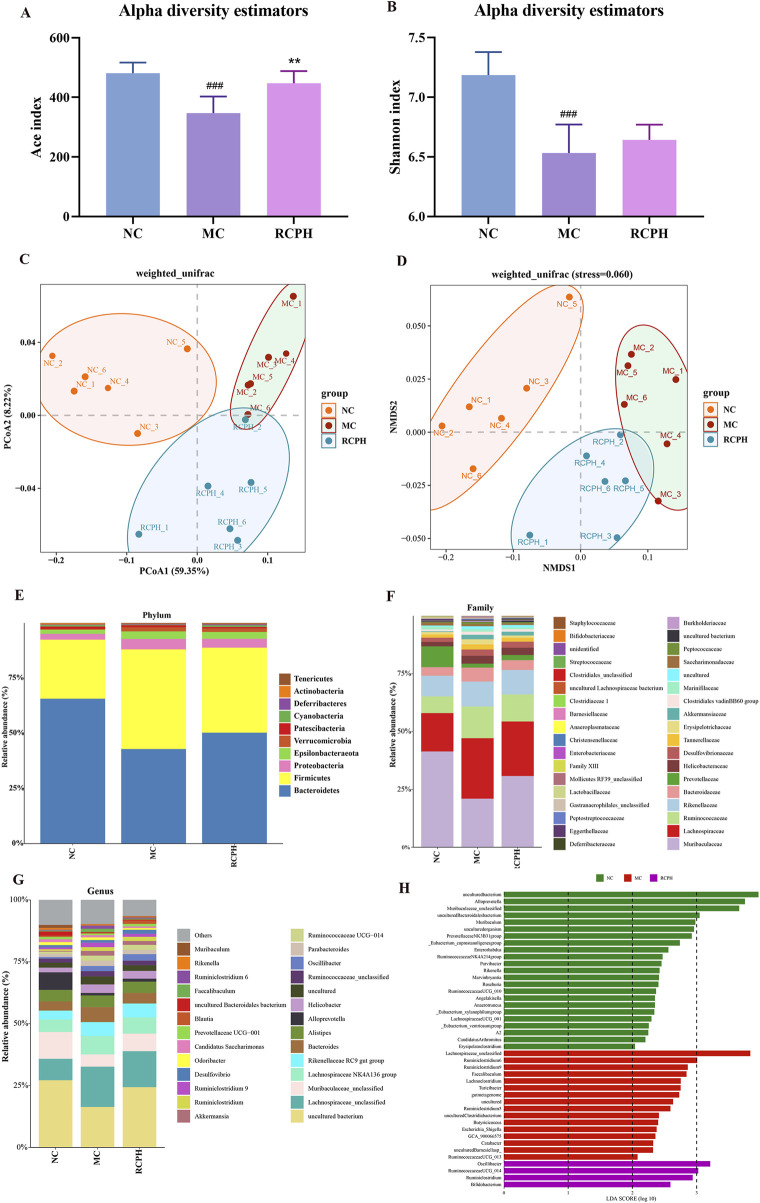
The impact of RCP on the gut microbiome of DSS-induced UC mice. **(A)** and **(B)** Alpha diversity analysis using the ACE and Shannon indices, respectively. **(C, D)** Beta diversity results from PCoA and NMDS analyses based on unweighted UniFrac distance matrices. **(E–G)** The distribution of intestinal microbiota at the phylum, family, and genus levels, respectively. **(H)** LDA analysis of bacterial differences at the genus level, LDA score >2 (*n* = 6).

To investigate the potential regulatory role of RCPH on intestinal microbiota, we conducted 16S rRNA sequencing on colonic content samples derived from mice. In our microbiome analysis, we obtained 1,326,461 high-quality 16S rRNA reads, with an average of 73,692 reads per sample (ranging from 34,996 to 151,050). To assess variations in microbial diversity between groups, we aligned sequences to evaluate alpha and beta diversity. Alpha diversity indices, encompassing species richness and evenness, were analyzed. Ace index means the richness of samples, and shannon index means the evenness of samples. DSS significantly reduced the Ace (Figure 7A) and Shannon (Figure 7B) indices, indicating decreased bacterial richness and diversity. The RCPH group exhibited significantly elevated colonic microbiota richness compared to the DSS group. Beta diversity, which measures differences in microbial community composition, was analyzed using principal coordinate analysis (PCoA) and non-metric multidimensional scaling (NMDS), offering insights into intergroup differences. Weighted UniFrac distances were employed for PCoA to assess beta diversity, revealing distinct microbial structures between MC and NC mice (Figure 7C). RCPH treatment in colitis mice significantly altered microbial composition, resembling NC mice more closely. NMDS analysis further corroborated these differences in intestinal flora (Figure 7D), highlighting the efficacy of RCPH in modulating microbial profiles.

**FIGURE 8:**
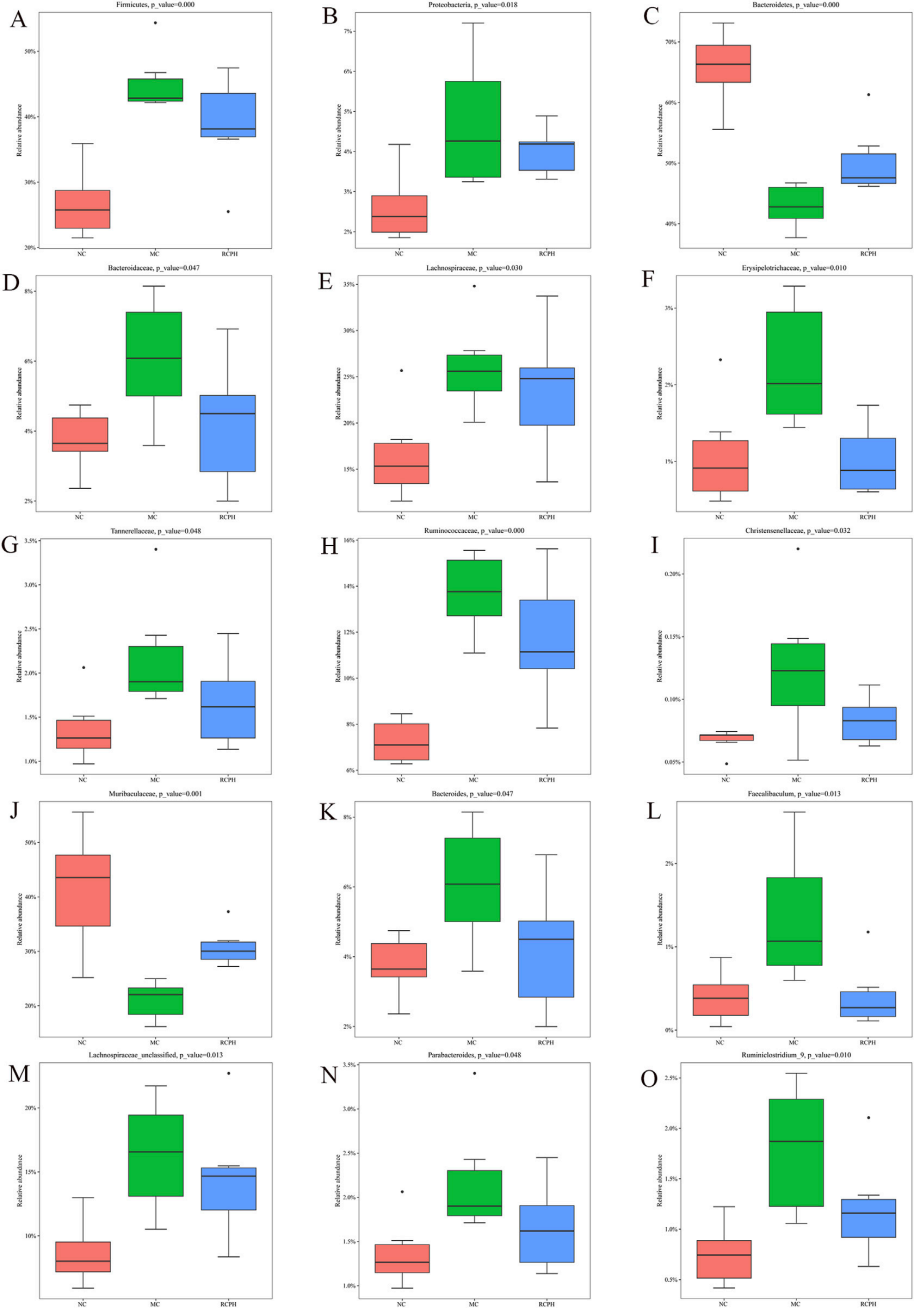
Main microbial differences at various levels. **(A–C)** Differences at the phylum level including Firmicutes, Proteobacteria and Bacteroidetes. **(D–J)** Differences at the family level including Bacteroidaceae, Lachnospiraceae, Erysipelotrichaceae, Tannerellaceae, Ruminococcaceae, Christensenellaceae and Muribaculaceae. **(K–O)** Differences at the genus level including *Bacteroides*, *Faecalibaculum*, *Lachnospiraceae_unclassified*, *Parabacteroides* and *Ruminiclostridium_9* (*n* = 6).

We analyzed microbial composition at various taxonomic levels (phylum, family, genus) to elucidate intestinal microbiota specifics. Key findings at the phylum level (Figure 7E) reveal ten major phyla, with Bacteroidetes, Firmicutes, and Proteobacteria dominating (>90% share), and inversely correlating Bacteroidetes abundance with GI inflammation. The model group exhibited a marked increase in Firmicutes and Proteobacteria, while Bacteroidetes levels were notably decreased. RCPH treatment restored the composition of Bacteroidetes phylum and decreased the Firmicutes in MC mice, which was similar to the NC mice (Figures 8A–C). The relative abundance of several families, including Bacteroidaceae, Lachnospiraceae, Erysipelotrichaceae, Tannerellaceae, Ruminococcaceae and Christensenellaceae were significant increased by DSS but reduced in the RCPH treatment group (Figures 8D–I). Another obvious change was observed in Muribaculaceae, MC mice showed a reduction in Muribaculaceae compared to NC mice, although levels of this bacterium were significantly higher in the RCPH group (Figure 8J).

The shifts in gut microbiota at the genus level were presented in Figures 8K–O. The relative abundance of *Bacteroides*, *Faecalibaculum*, *Lachnospiraceae_unclassified*, *Parabacteroides* and *Ruminiclostridium_9* were enriched by MC, but RCPH could inhibit these microorganisms. Using Linear Discriminant Analysis (LDA) Effect Size (LEfSe) analysis, we identified significant genus-level biomarkers that differentiate the various groups. Compared to the NC and MC groups, the RCPH group exhibited significant differences in *Oscillibacter*, *Ruminococcaceae_UCG_014*, *Ruminiclostridium*, and *Bifidobacterium* (Figure 7H). The results indicate that DSS caused disruption in the intestinal microbial community, and RCP intervention led to changes in the intestinal microbial community that made it more similar to the NC group.

### 3.6 RCPH treatment promoted the production of SCFAs

**FIGURE 9:**
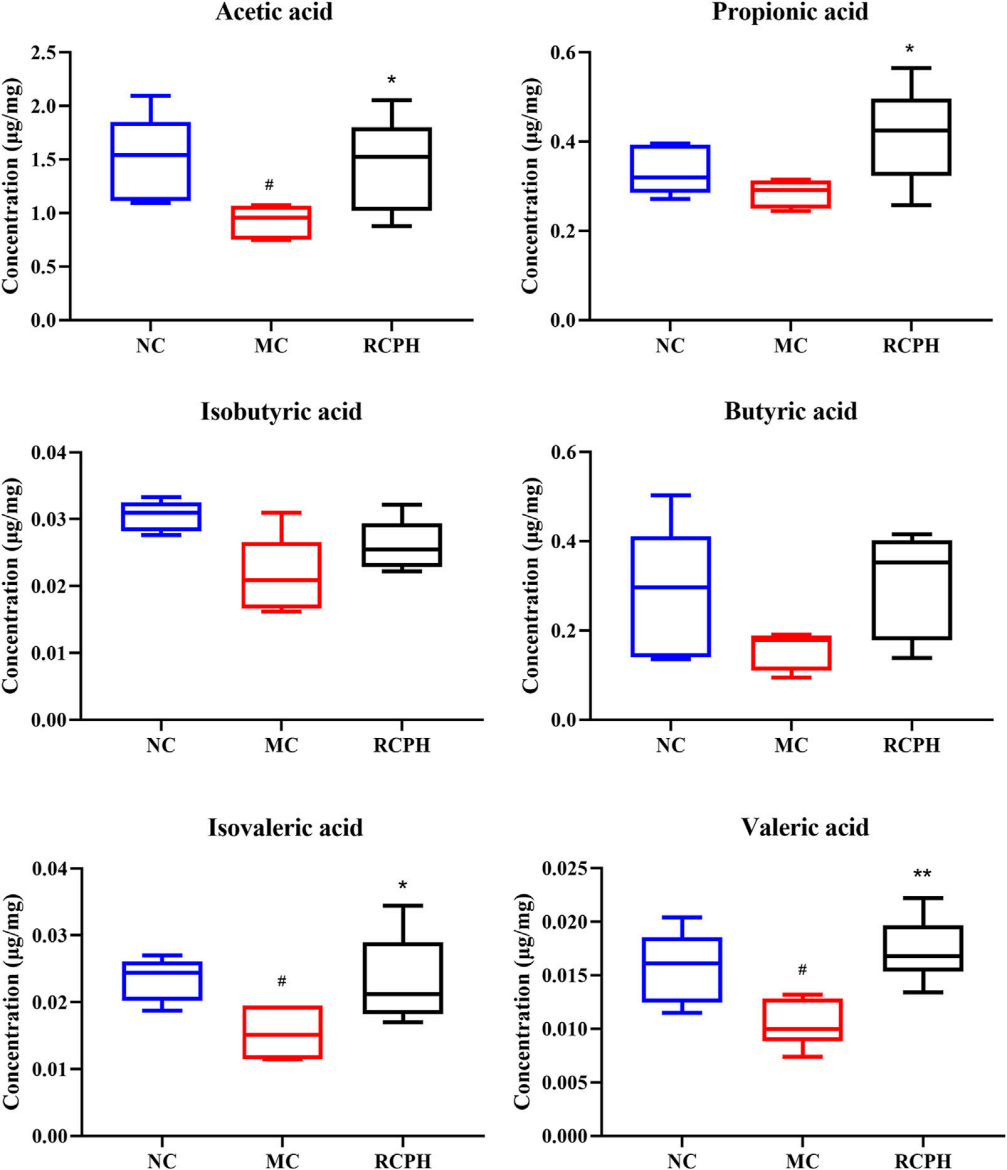
Effect of RCPH on SCFAs content of DSS-induced colitis in mice, including acetic acid, propionic acid, isobutyric acid, butyric acid, isovaleric acid and valeric acid. Data (*n* = 6 per group) are expressed as median (IQR); ^#^ *p* < 0.05, ^##^ *p* < 0.01 *versus* NC group; *p < 0.05, **p < 0.01 *versus* MC group.

The contents of SCFAs in cecal contents from each group were determined by GC-MS. As illustrated in Figure 9, DSS treatment notably altered the levels of SCFAs, significantly decreasing acetic, isovaleric, and valeric acids, while no significant differences were observed in propionic acid, isobutyric acid, and butyric acid levels between the groups. RCPH therapy reversed this, restoring acetic, propionic, isovaleric, and valeric acids to NC group levels, with butyric acid levels showing a recovery trend. RCPH can restore the stability of the intestinal microecology by modulating the levels of SCFAs.

### 3.7 Spearman correlation analysis

**FIGURE 10:**
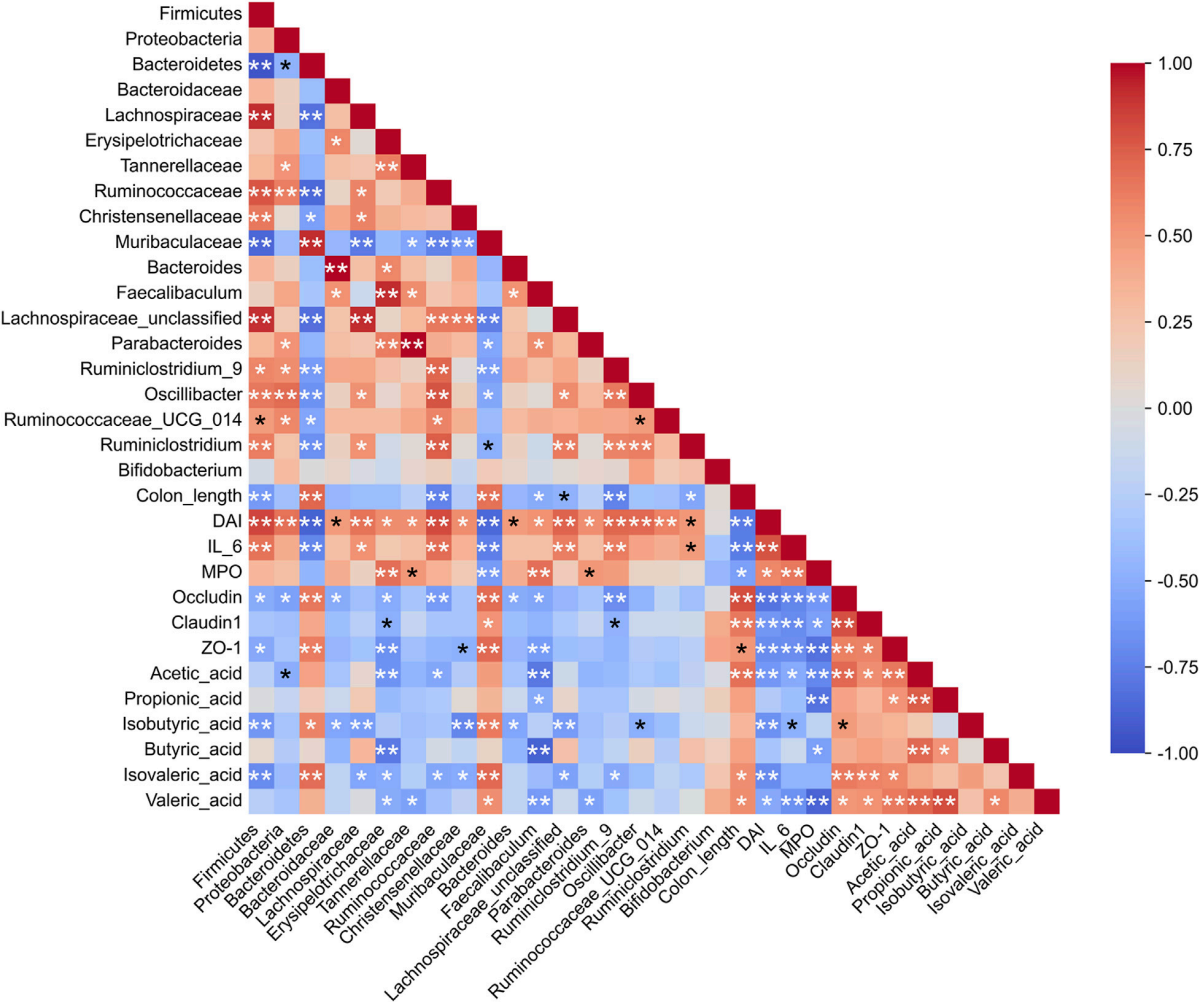
Spearman correlation analysis among gut microbiota, inflammation, tight junction proteins, and SCFAs. *p < 0.05, **p < 0.01.

To explore the relationships among key intestinal bacteria, biochemical markers, and SCFAs, Spearman’s correlation analysis was conducted. The findings revealed that the abundance of Muribaculaceae and Bacteroidetes showed a positive correlation with colon length, structural proteins, and various types of SCFAs, while it was negatively correlated with the DAI score and inflammation (Figure 10). Conversely, the abundances of Firmicutes, Lachnospiraceae, Erysipelotrichaceae, Tannerellaceae, Ruminococcaceae, *Faecalibaculum*, *Lachnospiraceae_unclassified*, *Parabacteroides*, *Ruminiclostridium_9*, and Ruminiclostridium exhibited a positive correlation with both the DAI score and the inflammation index. Furthermore, the abundances of Firmicutes, Ruminococcaceae, *Faecalibaculum*, *Lachnospiraceae_unclassified*, *Ruminiclostridium_9* exhibited a negative correlation with colon length and SCFAs. The correlations between *Oscillibacter*, *Ruminococcaceae_UCG_014*, *Ruminiclostridium*, and *Bifidobacterium* and the tested indicators were not significant, which may be related to polysaccharide degradation or other metabolic functions. Acetic acid and valeric acid exhibited negative correlations with DAI, IL-6, and MPO, while demonstrating positive correlations with the structural proteins occludin, claudin1, and ZO-1. These findings suggested that acetic acid and valeric acid played significant roles in the treatment of UC using RCP.

## 4 Discussion

RCP has various pharmacological activities including liver protection, frozen sperm protection, immune regulation, etc. However, its therapeutic activity against UC has not been systematically investigated. Our study demonstrates that RCP is effective in improving signs of disease and pathological damage, regulating the levels of inflammatory cytokines, inhibiting oxidative stress, increasing TJ proteins, maintaining the dynamic balance of intestinal microbiota and SCFAs, restoring the intestinal microecology in the DSS-induced UC mice.

In our preparation of RCP, we have employed membrane separation technology, which offers convenience and speed over traditional dialysis methods, while also eliminating the risk of contamination. Following treatment with D101 resin to remove pigments and proteins, the obtained RCP exhibits excellent solubility, stable molecular weight, and consistent sugar content. This process holds promise for scale-up production. In our follow-up work, we will focus on the isolation and purification of homogeneous polysaccharides to conduct in-depth studies on its spatial characteristics. The monosaccharide composition and proportions of RCP differ somewhat from those reported in existing research (Wu et al., 2023), potentially due to variations in the source of Rhodiola and differences in extraction and purification methods. However, they all contain Ara, GalA, Rha, and Gal. Notably, RCP contains 44% GalA and 26% Ara, which may constitute the primary pharmacological characteristics of RCP.

Immune dysfunction is the cause of many non-communicable human diseases, including autoimmune disorders, allergies, and cancer. The gastrointestinal tract serves as the main site where the host immune system interacts with symbiotic and pathogenic microorganisms. The disruption of bacterial microbiota leads to dysregulation of adaptive immune cells, which may be the basis for diseases such as inflammatory bowel disease (IBD). Maintaining a balance between anti-inflammatory and pro-inflammatory cytokines is crucial for the pathogenesis of IBD. Our results demonstrate that RCP could significantly ameliorate the dysregulation of pro-inflammatory cytokines IL-6 and MPO caused by colitis. Multiple studies have shown that in UC animal models, the expression levels of TNF-α, IL-6, and MPO increase, while there is a decline in anti-inflammatory cytokines such as IL-10 (Wang et al., 2019; Bai et al., 2020; Zou et al., 2020). As a multifunctional cytokine, IL-6 is regarded as a key contributor to inflammatory processes. It can exacerbate tissue inflammation and damage intestinal barriers by inhibiting T-cell apoptosis (Kishimoto, 2005), suggesting that RCP may resist UC by modulating immunity. The prevention and treatment of UC using polysaccharides is a complex process that may be regulated by one or more signaling pathways.

The intestinal barrier is composed of the integrity, tightness, and mucosal homeostasis of the intestinal epithelium. In the initial phases of ulcerative colitis, the primary etiology lies in the disruption of the intestinal epithelium, leading to the impairment of mucosal homeostasis (Neurath, 2014). As key regulators of paracellular permeability, TJ proteins play a pivotal role in maintaining intestinal permeability, tissue differentiation, and homeostasis. RCP treatment significantly increased the protein expression such as occludin, claudin1, and ZO-1 when compared to DSS stimulation. This is in agreement with prior reports that Scutellaria baicalensis Georgi polysaccharide (Cui et al., 2021), Angelica Sinensis Polysaccharide (Cheng et al., 2020) and Lycium barbarum Polysaccharide (Cao et al., 2021) ameliorate the reduction of TJ proteins in DSS-induced UC. Therefore, RCP enhances the expression of TJ proteins, which contributes to the repair of the intestinal barrier and improves its integrity and stability. This enhancement leads to a decrease in the permeability of harmful substances, reducing their damaging effects. Further investigation reveals that RCP has a significant therapeutic effect on UC, meriting in-depth research.

Disorders of the gut microbiota lead to the impairment of the intestinal barrier, resulting in the occurrence and progression of colitis. RCP restores the intestinal flora structure of UC model mice, bringing the ratio of Firmicutes to Bacteroidetes close to normal levels, reducing the numbers of *Bacteroides* and Parabacteroides to near-normal levels, while protecting beneficial bacteria such as Muribaculaceae and Bifidobacterium thereby shielding the intestinal mucosa from harmful bacteria. Previous studies have shown a decreased ratio of Firmicutes to Bacteroidetes in UC mice (Li et al., 2022; Li et al., 2024), and elevated levels of Proteobacteria in IBD patients compared to healthy individuals (Guo et al., 2021). These findings are consistent with our own observations. Notably, RCPH significantly increased the abundance of *Bifidobacterium*, a beneficial bacterium in both human and animal intestinal flora. Previous research has indicated that *Bifidobacterium* can help regulate gut flora and exhibits strong therapeutic potential for intestinal inflammation (Dong et al., 2022). Studies have shown that Muribaculaceae are beneficial bacteria that play a crucial role in polysaccharide degradation (Qiu et al., 2024), whereas *Faecalibaculum* is associated with UC and exhibits a pro-inflammatory effect (Luo et al., 2024). Importantly, RCP enhanced the abundance of Muribaculaceae while decreasing the levels of *Faecalibaculum*, which contributed to reduced inflammation and the restoration of intestinal homeostasis. A ketogenic diet consisting of a high proportion of fat and low carbohydrates aggravates DSS-induced UC (Li et al., 2021). A significant portion of the human gut microbiota’s genome is dedicated to the degradation and absorption of carbohydrates (Wardman et al., 2022). This also indicates the importance of supplementing polysaccharides. Although we have demonstrated the role of RCP in modulating the gut microbiota, the specific microbiota that break down and utilize RCP have not been clearly identified. Understanding these key microbiota would be beneficial in elucidating the metabolic process of RCP *in vivo* and warrants further investigation.

SCFAs are crucial for intestinal and immune balance. While bacteria degrade carbohydrates for their own nourishment, they provide SCFAs to the host, contributing up to 10% of the body’s energy needs. These SCFAs additionally exhibit anti-inflammatory effects, regulate epigenetic changes, and impact host metabolic processes (Koh et al., 2016). Acetate, the most prevalent SCFA in the peripheral circulation (Cummings et al., 1987), is capable of crossing the blood-brain barrier, thereby reducing appetite through central homeostatic mechanisms (Frost et al., 2014). Acetic acid is the primary fermentation metabolite produced by the majority of bacteria (Parada Venegas et al., 2019). Studies have shown that valeric acid consistently exhibits a negative correlation with the severity of UC (Liu et al., 2024), which aligns with our findings. RCP not only serves as a nutrient source for the intestinal microbiota but also, through the metabolic activity of these microbiota, produces beneficial SCFAs, particularly acetic acid and valeric acid. These fatty acids bolster epithelial barrier function *via* signal transduction, suppressing pro-inflammatory factors to uphold intestinal wellness. The polysaccharides derived from plants possess a remarkable capability to enhance the abundance of probiotics, notably including SCFAs producing bacteria such as *Lactobacillus*, *Roseburia* and *Bifidobacteria*. These SCFAs play a pivotal role in sustaining the colonic milieu’s equilibrium, fostering the growth of intestinal epithelial cells, stimulating immune responses, and optimizing intestinal barrier functionality, thereby contributing significantly to gut health.

These results suggest that RCP may improve UC by regulating intestinal microbiota and metabolic product SCFAs, to restore intestinal barrier function. Furthermore, these findings indicate that RCP could serve as a promising candidate for new therapeutic options for patients with UC.

## 5 Conclusion

In summary, RCP significantly treated DSS-induced colitis in mice, through strengthening intestinal barrier integrity, modulating cytokine levels, and improving the composition of gut microbiota along with SCFA levels. Collectively, these multifaceted benefits significantly contributed to the alleviation of colitis symptoms. We are confident that the findings of this study offer the potential to provide a research foundation for the drug development and clinical treatment of Rhodiola polysaccharides. However, there is still room for further research on the purification and characterization of RCP as well as its relationship with *Bifidobacterium*, which can lay a foundation for delving into its preparation and mechanism of action.

## Data Availability

The data presented in the study are deposited in the NCBI repository, accession number PRJNA1222194.
